# C-reactive protein point-of-care testing in children with cough: qualitative study of GPs' perceptions

**DOI:** 10.3399/bjgpopen17X101193

**Published:** 2017-10-27

**Authors:** Marjolein JC Schot, Berna DL Broekhuizen, Jochen WL Cals, Esther Brussee, Niek J de Wit, Theo JM Verheij, Esther de Groot

**Affiliations:** 1 GP, PhD Student, Julius Center for Health Sciences and Primary Care, University Medical Center Utrecht, Utrecht, The Netherlands; 2 GP, Postdoctoral Researcher, Julius Center for Health Sciences and Primary Care, University Medical Center Utrecht, Utrecht, The Netherlands; 3 GP, Postdoctoral Researcher, Department of Family Medicine, CAPHRI School for Public Health and Primary Care, Maastricht University, Maastricht, The Netherlands; 4 Senior House Officer, Julius Center for Health Sciences and Primary Care, University Medical Center Utrecht, Utrecht, The Netherlands; 5 GP, Professor of Primary Care, Julius Center for Health Sciences and Primary Care, University Medical Center Utrecht, Utrecht, The Netherlands; 6 GP, Professor of Primary Care, Julius Center for Health Sciences and Primary Care, University Medical Center Utrecht, Utrecht, The Netherlands; 7 Postdoctoral Researcher, Julius Center for Health Sciences and Primary Care, University Medical Center Utrecht, Utrecht, The Netherlands

**Keywords:** child, C-reactive protein, point-of-care testing, respiratory tract infections, primary health care, qualitative research

## Abstract

**Background:**

Point-of-care C-reactive protein (CRP) testing is widely accepted in Dutch general practice for adult patients with acute cough, but GPs’ perceptions of its use in children with suspected lower respiratory tract infection (LRTI) are unknown. Knowledge of these perceptions is important when considering broadening its indication to use in children.

**Aim:**

To explore the perceptions of Dutch GPs of the addition of point-of-care CRP testing to the diagnostic evaluation of children, and compare these to their perceptions of use in adults.

**Design & setting:**

A qualitative study in general practice in the Netherlands.

**Method:**

Semi-structured interviews were held with 11 GPs. Interviews were analysed using open coding and a thematic approach.

**Results:**

GPs’ perceptions of the addition of point-of-care CRP testing to diagnostic process in children with suspected LRTI differ from their perceptions of this in adults. Five themes were identified: patient characteristics; vulnerability of the child; clinical presentation; availability of evidence; the impact of the procedure; and use of point-of-care CRP testing as a communication tool.

**Conclusion:**

Differences between the perceptions of using point-of-care CRP testing in children and adults need to be addressed when considering the possible implementation of this diagnostic instrument.

## How this fits in

Point-of-care CRP measurement enhances GPs’ confidence in prescribing decisions for adults with acute cough and helps GPs to manage adult patients’ expectations for antibiotics. This qualitative study shows that GPs’ perceptions of adding point-of-care CRP testing in children with acute cough differ from their perceptions of use in adults. This was apparent over five themes: the perception of a child as a vulnerable patient; the difference in clinical presentation between adults and children; the impact of a finger prick on a child; the lack of evidence on the use of point-of-care CRP testing in children; and the use of point-of-care CRP testing as a tool for communication.

## Background

Point-of-care testing (POCT) CRP has proven to safely reduce antibiotic prescriptions for adults with acute cough^1^ and has been incorporated in the National Institute For Health and Care Excellence (NICE) guideline on community-acquired pneumonia^2^ and in the Dutch primary care practice guideline.^3^ Acute infections in children are common, with acute respiratory tract infection being the most common diagnosis in primary care. LRTI has an annual incidence of 34.3 per 1000 children.^4^ Antibiotic overprescription in children is a problem, as it is in adults, with inappropriate prescribing occurring in 32% of consultations for children with LRTI.^5^ Whether the use of POCT CRP for children with suspected LRTI is effective in safely reducing antibiotic prescriptions is currently under investigation. Dutch national guidelines discourage its use in children until further research provides evidence on its clinical value.^3^ If proven effective, knowledge of GPs’ perceptions of the use of POCT CRP in children with acute cough is essential as uptake of a new diagnostic instrument is highly dependent on the perceptions of users.^6,7^ Although sometimes overlooked, this aspect may be as important as an assessment of the effectiveness of the diagnostic intervention itself.

Qualitative studies show that for adult patients, POCT CRP enhances patients' and GPs’ confidence in prescribing decisions as it supports the diagnostic and therapeutic process and helps GPs to manage patients’ expectations for antibiotic treatment for LRTI.^8,9^ GPs mentioned disadvantages such as difficulties with the interpretation of test results and possible distraction from clinical reasoning when using a diagnostic device.^9^ One pilot study evaluated the acceptability and usability of POCT CRP in children and found that parents would likely accept the test, but the five GPs interviewed were divided on the utility of the diagnostic test.^10^

Currently, half of all Dutch GPs and 15% of UK GPs have POCT CRP available in their practice.^11^ It is unknown how GPs with ready access to and experience with POCT CRP in adults view the possible introduction of this test in children. There are, however, reasons to believe that their evaluation might be different, as children are considered a vulnerable group of patients.^12^

This study aims to understand why GPs in the Netherlands would decide to or decide not to include POCT CRP measurement in the diagnostic process in children with LRTI, especially in comparison to their perceptions of using POCT CRP in adults. The study, therefore, aims to answer the following research question: do GPs’ perceptions of the use of POCT CRP in the diagnostic evaluation of children with LRTI differ from their perceptions of the use of this test in adults and, if so, in what respect?

## Method

This qualitative study was performed as part of a broader research programme, which also includes an ongoing pragmatic randomised controlled trial concerning the effect of POCT CRP measurement in children with suspected LRTI on antibiotic prescriptions (PRICE, Dutch trial registration code 4399). In this trial, GPs in the intervention arm are instructed to use POCT CRP when they experience diagnostic uncertainty in children with suspected LRTI.

GPs were approached to participate in this study first based on convenience sampling: three GPs from the authors' clinical network and three GPs, with varying clinical experience, participating in the aforementioned trial were interviewed. Thereafter, purposive sampling was applied to recruit other GPs to ensure that the study incorporated the views of GPs who were known advocates and opponents of the introduction of POCT CRP in adults, academic GPs, and GPs with differing, subject-relevant clinical experience. Participants provided written informed consent to participate in this study.

Participants were interviewed face to face in their own surgery using a topic guide (Box 1). Interviews were centred around the following topics: the GP’s opinion on the use of POCT CRP in children with suspected LRTI; the factors influencing the GP’s decision to add POCT CRP to the diagnostic evaluation of children; and the possible consequences of the use of POCT CRP in children with LRTI. The topics functioned as a guide during the interviews without being restrictive and thus allowed the flexibility to explore emerging themes. As data collection and analysis were carried out iteratively, the topic guide was adapted as the interview process progressed based on new insights gained. The same interviewer conducted all interviews. Interviews took place between January and May 2015, lasted about 30 minutes, were fully audiorecorded, and were transcribed verbatim before analysis. As a member check, a summary of the interviews was sent to the GPs, but this did not lead to adjustment of any of the transcriptions. When, after convenience and later purposive sampling, no new perceptions were voiced and no new codes emerged during analysis, it was decided that data saturation had been reached and the conducting of additional interviews stopped.

**Box 1.** Topic guide for interviews with GPs

1. What is your opinion on the use of POCT CRP in children with LRTI in primary care?
2. Which factors influence you in your decision (not) to add POCT CRP to the diagnostic evaluation of children with LRTI?
3. What are, in your opinion, possible consequences of the use of POCT CRP in children with LRTI in primary care?

The anonymised transcripts were analysed in five steps: familiarisation; open coding; creation and revision of categories and subcategories; abstraction; and interpretation.^13^ Familiarisation started with reading each interview at least once. Transcripts were then reread and coded where they contained concepts related to the study's research question. When new codes emerged during the process, preceding transcripts were reread and recoded where needed. Subsequently, categories and subcategories were derived from initial codings. Abstraction refers to the generation of main categories by grouping subcategories together as much as possible. Eventually, results were interpreted into themes to formulate an answer to the study's research question.

The study team consisted of researchers with different backgrounds. One has a background in the learning sciences, and all other researchers have a background in primary care research and work in general practice. All researchers who did the analysis have experience with qualitative research. The different backgrounds allowed complementary views on the collected data. Reliability was endorsed by coding all data by two researchers. Discrepancies in coding or interpretation were resolved through discussion. Categories were set up after discussion and agreement between researchers. All interview transcripts were managed and analysed using NVivo 10.0.

## Results

### Characteristics of responders

Eleven GPs from nine general practices participated. All participants had experience with CRP as a laboratory biomarker, eight participants had access to POCT CRP in their practice, and three GPs were participating in the ongoing randomised clinical trial. Most GPs used POCT in adults regularly. Only one GP did not use CRP measurement in the diagnostic process of LRTI in adults. GPs stated that they never or rarely used POCT CRP in children. Even the three GPs that were involved in the trial indicated they rarely used the test in children.

GPs mentioned multiple differences between adults and children concerning the possible addition of POCT CRP in a diagnostic process. Five themes were identified in the data: patient characteristics; clinical presentation; availability of evidence; the impact of the procedure; and the use of POCT CRP as a tool for communication.

### Patient characteristics: vulnerability of the child

GPs mentioned considering children as more vulnerable than adult patients. This was illustrated in several ways: they mentioned their fear of unpredictable and faster deterioration in children with LRTI, but also feared that, in cases of such sudden deterioration, this might not be noticed in a timely manner by some parents. Many mentioned not wanting to miss a serious infection or risk complications specifically in children:

*‘In adults … at least they feel it themselves, it’s their own body, and they will call again if necessary. An adult can also stand more. But a small child … yes, it can also change very quickly. And in adults, you just don’t see that very often.’* (GP11)

*‘Young children are more vulnerable than adults, for whom you can use watchful waiting … in adults it’s easier to say: "we will wait and if you don’t improve in a day or two you can come back: … In children, this is possible too, but you’re more cautious. You don’t want to take the risk that the child deteriorates and that it could have been prevented with antibiotics.’* (GP10)

GPs mentioned being more cautious, leading to quicker antibiotic prescription for children than for adults. This cautious approach also influenced their appraisal of POCT CRP. Several GPs stated that their clinical assessment would not be affected by a low POCT CRP test result in the event of diagnostic uncertainty in children, as it would in adults:

*‘In children, I might be a bit more cautious, so then I tend to prescribe antibiotics, yes, when in doubt, and skip the POCT CRP.’* (GP2)

### Clinical presentation

Patients are evaluated based on history and physical examination by a GP. GPs agreed that presentation of symptoms could be quite different in adults as compared to children. Most mentioned that, even though history taking is more difficult in children, assessment of the severity of their disease is more straightforward than with adults.

*‘And you know, a child is, I would almost say, purer in a sense. A child feels ill or it doesn’t and you can see that. An adult can make all sort of theatre around this and, yes, sometimes you will then think "erm* ... *it is not that bad". Or the other way around.’* (GP1)

It seemed that GPs less often felt uncertain about their judgement of a child than of an adult, which led them to conclude that the addition of POCT CRP was not needed.

### Availability of evidence

GPs mentioned another obstacle in using POCT CRP in children: whereas the use in adults is recommended by professional guidelines, two GPs specifically mentioned that since guidelines currently do not recommend the use in children, this prevented them from using the test. Others mentioned that they considered the present level of evidence for using POCT CRP in children insufficient to justify adding it to the diagnostic process. A majority mentioned they had difficulty with the interpretation of test results and were unsure of optimal cut-off values for children. They specified that it was unclear whether the cut-off values that were recommended by guidelines for adults could also be applied to children. Some indicated that, because of this lack of clarity on cut-off-values and lack of evidence with regard to the trustworthiness of test results, they valued their clinical judgement over the CRP values, which led them to skip CRP measurement or decide to prescribe antibiotics even when CRP values were low:

*‘I just don’t know what the normal range is … you know, in adults we stick to 20 and 100, that is the range, those are the limits. And I don’t know whether those values also apply in children.’* (GP4)

*‘At this stage, there is no clear evidence that POCT CRP is a useful tool in children, so, I actually don’t use it at all.’* (GP8)

### Impact of the procedure

The majority of GPs considered the required finger prick to collect the blood sample for testing to be invasive. They were reluctant to hurt or scare their young patients, or cause fear of future consultations. In contrast, in adults, the blood sampling procedure itself was not a reason to refrain from POCT CRP. GPs expressed they could easily explain the necessity of the test and the required finger prick to adults, whereas children often would not understand this explanation:

*‘… I try to perform as few painful examinations in children as possible to avoid children being scared during future visits to my surgery. I would really like to keep a good relationship with the child and by performing painful pricks well … it can create reluctance during future consultations, this in contrast to adults where you can easily explain, and this is not an issue.’* (GP5)

### Use of POCT CRP as a communication tool

GPs noted that they not only used POCT CRP in adults in case of their diagnostic uncertainty, but also as a tool to communicate a non-prescription decision. GPs indicated they seldom used POCT CRP in children to convince parents that antibiotics were not necessary. Many pointed out that, in their perception, parents quite often had reservations about giving their children antibiotics, whereas GPs often felt pressure from adult patients to prescribe. However, some GPs did mention there might be situations, although rare, in which POCT CRP could be used in children to reassure parents that antibiotics are not necessary:

*‘If parents are really worried, but after physical examination, I myself am reassured, I will try to explain this to the parents. In one case I did use POCT CRP because I could not reassure the parents.* […] *This is an exception. I actually never use it, in children, for this reason.’* (GP2)

The three GPs without access to POCT CRP had similar arguments against the use of POCT CRP in children as those who did have access to the test. However, they were more outspoken in their perceptions. They also stressed the importance of clinical assessment over the use of additional tests and indicated that they felt it would never influence their clinical judgement and would not change their antibiotic prescription policy. All three pointed out that the current level of evidence had not convinced them POCT CRP would have additional value in children. In contrast to the other GPs, two of them felt this also to be true for adults.

## Discussion

### Summary

In a primary care setting in which most GPs have access to and experience with POCT CRP, GPs’ perceptions of adding POCT CRP to the diagnostic process of LRTI in children are quite different from their perceptions for adults. While GPs feel that POCT CRP is helpful in managing adults with a cough in case of diagnostic uncertainty and in communicating about their decision not to prescribe antibiotics, they are quite reluctant to add POCT CRP to the diagnostic evaluation of children with LRTI. Themes identified were the vulnerability of a child as a patient and the differences in clinical presentation between children and adults. Furthermore, the lack of evidence for the use of POCT CRP in children was a theme, as was the invasiveness of the diagnostic test. The last theme was the use of POCT CRP as a tool for communicating, which was also viewed differently for children.

GPs express that their current management of children with LRTI is primarily based on an assessment of clinical symptoms and is characterised by a more cautious approach than in adults, where they are less inclined to watchful waiting. Specifically, and in contrast with adult patients, GPs fear fast and unexpected clinical deterioration of symptoms in children, which makes them apply a ‘better safe than sorry’ approach in prescribing decisions. They view the reduction of diagnostic uncertainty by POCT CRP as less important in children than in adults. In combination with uncertainty towards the exact diagnostic value of POCT CRP and usable cut-off values, this leads to reservations about the implementation of POCT CRP for children.

Various GPs mention they frequently use POCT CRP in adults to convince patients about the low severity of disease and to support their non-prescribing decisions. GPs express that they do not need this in children, as they feel less pressured by parents to prescribe antibiotics, and parents understand non-prescribing decisions.

### Strengths and limitations

This study is the first in-depth qualitative study exploring GPs’ perceptions of POCT CRP in children with LRTI in a setting where most GPs have access to and experience with this diagnostic instrument. The chosen sampling strategy enabled us to explore and compare GPs’ perceptions about the research topic. Double coding of all transcripts and member checks secured reliability. An inductive approach, with the addition of interviews until data saturation, allowed themes to emerge from the data rather than being established before data collection and analysis.

The perceptions of Dutch GPs interviewed in this study do not necessarily mirror those of GPs in other countries, as country-specific health systems and cultural factors may influence GPs’ perceptions of the implementation of tests such as POCT CRP. However, in earlier studies, GPs’ perceptions of the implementation of POCT CRP for adults did not differ much across countries with a strong primary care system.^9,11,14^

### Comparison with existing literature

It is understood to be the case that only one mixed methods study has previously investigated the acceptability and usability of POCT CRP in children in a primary care setting in the UK.^10^ However, the five participating GPs in that study had little or no experience with this diagnostic instrument. Acceptability was evaluated in parents, but not in GPs, as the finger prick was carried out before clinical assessment of the child. GPs were only asked if they found the addition of POCT CRP to their evaluation usable. This is a distinctly different procedure than the current practice in the Netherlands, where additional testing is only applied after clinical evaluation. The procedure of carrying out the finger prick before examination by the GP probably led to the fact that the UK-based study did not identify the reluctance to perform a finger prick by GPs as a theme, whereas this study found this to be a barrier to the use of POCT CRP. The need for more evidence and clear guidance regarding the diagnostic value of POCT CRP in children is a theme found in both studies.

It is known that medical professionals view children as a vulnerable group^12^ and that these beliefs, combined with perceived uncertainty in identifying children with a potentially serious bacterial infection, influence their prescribing decisions.^15^ A recent systematic review of qualitative studies examined views of clinicians on prescribing decisions in children.^16^ Clinicians reported prescribing antibiotics to children with acute infections ‘just in case’ when they were not confident about the diagnosis or about possible social, health, or legal consequences of not prescribing. Doubts about whether parents could safely monitor the illness, especially when patients were not familiar to the physician, were also reported to drive the prescription of antibiotics. This study aligned with this overall perception of children as a vulnerable group, with GPs not wanting to take the risk of a child deteriorating, and with their uncertainty about the parental role in the follow-up of children's suspected LRTI.

### Implications for research and practice

Whether POCT CRP can increase the diagnostic certainty of GPs and help to identify children who need antibiotics and those who do not is still under investigation. However, uptake of a diagnostic instrument and the impact of the test results on management depend on the individual GP.^17^ Therefore, it is important to further explore this study's finding that GPs reported less diagnostic uncertainty in children, as this seems to be one of the reasons that GPs involved in this study considered additional (invasive) diagnostic tests less necessary for the evaluation of children than for the evaluation of adults.

In conclusion, GPs’ perceptions of the use of POCT CRP in children differ from those of the use in adults with suspected LRTI. Uncertainty about the exact role of POCT CRP and relevant cut-off values, in combination with the perception of a child as a vulnerable patient, causes GPs to refrain from using the test at present. If POCT CRP proves to be useful in safely reducing antibiotic prescription in children, it is crucial for successful implementation that these perceptions are taken into account.
